# Miscellaneous Medical Intelligence

**Published:** 1849-01

**Authors:** 


### ARTICLE IV.

### MISCELLANEOUS MEDICAL INTELLIGENCE.

Lyne Starling, Esq., has added $5000 to his former donation of $30,000, to the Starling Medical College, at Columbus, Ohio.

The Legislature of Louisiana has appropriated thirty thousand dollars to the State University, most of which is to be spent on the medical department at New Orleans. We hope the citizens of Louisiana may always have scientific medical attendance when sick.

Dr. Meeker, of Laporte, Ind., reports a case in which a patient was poisoned to death in consequence of the application of 100 grains of corrosive sublimate to a blistered surface by a *cancer doctor*, to cure cancer of the breast. The quack explained the case by saying that the roots of the cancer reached the heart, and the medicine followed them there.

Gutta percha ʒj chloroform f℥j make a solution, which when applied to the clean, dry cavity of a carious tooth, cures the pain, and protects the tooth from decay.

The local application of anæsthetic agents has attracted considerable attention recently, but not with uniformly satisfactory results. Ice applied to a part until numbness is produced, seems to be the best local anæsthetic.

Dr. Lee, the accomplished editor of the New York Medical Journal has retired, and Dr. Purple succeeds him. Dr. Roberts, the able and enterprizing founder and editor of the Annalist, has handed his editorial quill over to "the young man of Binghampton," Dr. N. S. Davis, the indefatigable mover in founding the American Medical Association. Our excellent exchange, the Western Lancet, comes to us filled almost entirely with original matter.

The Alleghanians.—These far famed singers, under the direction of Mr. Brockelbank, whose skill in the management of such matters is unrivalled, gave a concert on New Year's evening for the benefit of the sick poor of Chicago—he says mainly to be applied to sending patients to the hospital. They had, we are glad to say, a very full house.

